# Chalcogenide Glass-Capped Fiber-Optic Sensor for Real-Time Temperature Monitoring in Extreme Environments

**DOI:** 10.3390/s21051616

**Published:** 2021-02-25

**Authors:** Bahareh Badamchi, Al-Amin Ahmed Simon, Maria Mitkova, Harish Subbaraman

**Affiliations:** Department of Electrical and Computer Engineering, Boise State University, Boise, ID 83725, USA; baharehbadamchi@u.boisestate.edu (B.B.); alaminahmedsimon@u.boisestate.edu (A.-A.A.S.); mariamitkova@boisestate.edu (M.M.)

**Keywords:** chalcogenide glass, optical fiber, temperature sensor

## Abstract

We demonstrate a novel chalcogenide glass (ChG)-capped optical fiber temperature sensor capable of operating within harsh environment. The sensor architecture utilizes the heat-induced phase change (amorphous-to-crystalline) property of ChGs, which rapidly (80–100 ns) changes the optical properties of the material. The sensor response to temperature variation around the phase change of the ChG cap at the tip of the fiber provides abrupt changes in the reflected power intensity. This temperature is indicative of the temperature at the sensing node. We present the sensing performance of six different compositions of ChGs and a method to interpret the temperature profile between 440 °C and 600 °C in real-time using an array structure. The unique radiation-hardness property of ChGs makes the devices compatible with high-temperature and high-radiation environments, such as monitoring the cladding temperature of Light Water (LWR) or Sodium-cooled Fast (SFR) reactors.

## 1. Introduction

Generation IV nuclear power plants operate at high temperatures to achieve higher efficiency [1,2,3]. Precise temperature monitoring within a nuclear reactor is critical for the stability, long-term reliability, and proper functionality of the reactor over its operational lifetime. Additionally, during the research and development stages of new reactor materials, structural components, and fuels, it is imperative to study their performance under specific test conditions before commissioning these materials or components. Such testing procedures require real-time temperature monitoring with high precision. So far, this has been a big challenge due to radiation–material interaction at high temperatures. Usually, in any high-radiation environment (e.g., reactor cladding, and spent fuel pool), the temperature is monitored using metal-based sensors like thermocouples, Resistance Temperature Detectors (RTD), or melt-wire sensors [4,5]. Although RTDs give real-time data about temperature, RTDs need external current to measure the resistance change. Thus, with measuring current I, I${}^{2}$R heating inside the RTD device presents itself as a problem known as “self-heating” [5]. Moreover, gamma heating due to radiation absorption gives erroneous reading. Melt-wires are inexpensive but do not allow for real-time monitoring of temperature and can only be checked post factum. Compared to melt-wires, thermocouples provide real-time reading of temperature. However, the output signal from a thermocouple is weak, has low resolution, and is highly sensitive to common mode noises, which compromises their accuracy. Additionally, the sensor performance is affected by oxidation and the temperature readings drift significantly under long-duration exposure to high temperature and radiation [6,7]. This often necessitates sensor recalibration due to transmutation from absorption of neutrons [4].

To overcome these limitations, optical fiber sensors (OFS) have emerged as an important candidate for monitoring different parameters, such as temperature, pressure, strain, and condition of coal-fired power plants within harsh environments [8,9,10]. Radiation-hard OFSs that possess low degradation and stability under harsh environment are the best candidates for temperature monitoring in spaces where conventional sensors such as melt-wires and thermocouples are not well suited [11,12,13]. OFSs offer several advantages over their electrical counterparts, such as compact size, low-cost, radiation tolerance, immunity against electromagnetic interferences (EMI), and low-loss signal transmission from within the nuclear reactor to an external control center [14,15,16]. Herein, we propose a novel, accurate, real-time temperature monitoring device through integrating chalcogenide glasses (ChG) with optical fibers, whose operation is based on the phase change of chalcogenide glasses at specific, well-defined temperatures.

ChGs are inorganic, disordered materials containing one or more of the chalcogen elements (group 16 of the periodic table), such as sulfur, selenium, and tellurium except for oxygen. ChGs can be synthesized by mixing the chalcogen elements with other elements of the periodic table, for example, arsenic, germanium, antimony, gallium, silicon, and phosphorus. These glasses with compositional flexibility also demonstrate ultrafast crystallization rate and large optical properties contrast between their amorphous (disordered) and crystalline (ordered) phase, making them ideal for infrared photonic applications [11,12,13,14,15,16,17,18,19]. They are easy to synthesize in bulk and in thin-film forms. When ChGs are heated, there are three temperatures of significant consideration which are related to change in material’s property: (1) glass transition temperature (T${}_{g}$), (2) crystallization onset temperature (T${}_{o}$), and (3) glass’ peak crystallization temperature (T${}_{c}$). Glass transition temperature (T${}_{g}$) is related to the onset of fluidity. At this temperature, the compositional network is broken and the building blocks are macroscopically mobile. Crystallization onset temperature (T${}_{o}$) or critical transition temperature is the temperature at which ChG begins phase transition from a glassy to a crystalline phase. Non-isothermal crystallization appears as an exothermic Gaussian curve in the Differential Scanning Calorimetry (DSC) results, and the corresponding temperature at the peak of that curve is called the peak crystallization temperature (T${}_{c}$). It is specific for each material composition. At the onset of crystallization, the nucleation process is fast and switching occurs within 80–100 ns [18,19,20,21], and this transition changes the material’s conductivity, refractive index, absorption, and extinction coefficient. The resulting crystalline phase exhibits excellent conductive characteristic. This characteristic has led to several emerging phase change applications [22,23,24]. By creating a suitable thermodynamic conditions, the crystalline material can be amorphized back [20] and all initial properties of the ChG can be restored. Additionally, ChGs are radiation hard due to the lack of long-range order and the presence of lone pair p-shell electrons. ChGs inherently contain a high number of defects [25]. These defects rapidly recombine with the defects generated by the irradiation, thus stabilizing the structure and properties [25,26]. The effect of irradiation on chalcogenide glasses has been widely studied [27,28,29,30,31,32] and their radiation hardness has been well documented [27].

The sensors presented here have a structure based on a temperature sensitive ChG cap fabricated on the end facet of a radiation-hard gold-coated single-mode fiber (SMF). The working principle of the sensor is based on the big observable difference in the optical power reflected from the fiber-ChG interface as the ChG undergoes transition from the amorphous to the crystalline phase, around T${}_{c}$. The phase transition affects the Fresnel reflection intensity at the interface between the optical fiber facet and the ChG-capped fiber and so carries information about the temperature at the sensor node. The proposed device is simulated and its performance characteristics are experimentally verified. In order to confirm reliability of the device, the operation of the temperature sensors fabricated with two ChG material families, Ge${}_{x}$${\mathrm{Se}}_{100-x}$ and Ge${}_{x}$$S_{100-x}$ (x = 30, 33, 40) glasses are experimentally demonstrated. Two different methods are applied for device fabrication, i.e., ChG cap formation, (1) Thermal Evaporation and (2) Dip-coating in nanoparticle ink. Therefore, based on fabrication method and composition, in total 12 different types of devices were tested. The experimental results are in good agreement with the simulations. A novel temperature sensor architecture for monitoring temperature evolution in real-time in extreme environments through organizing these single sensors with different ChG caps deposited in an array structure, is also presented. Due to the compactness of the sensor tips, a sensor array head active area of 2.125 mm${}^{2}$ can be achieved for temperature monitoring with resolution 0.4–2 °C over 440 °C and 600 °C. These temperatures are important for the cladding temperature monitoring of LightWater (LWR) or Sodium-cooled Fast (SFR) reactors.

## 2. Sensor Design

**Figure 1:**
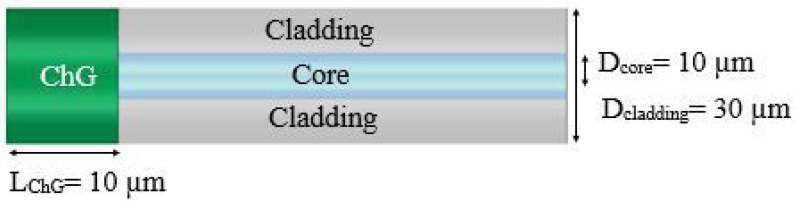
Schematic cross section of proposed ChG-capped optical fiber tip-based temperature sensor.

The proposed optical fiber-based temperature sensor architecture is shown in Figure 1. It has been obtained by ChG deposition by vacuum evaporation or dipping in ChG ink of the cleaned from the gold cover and cleaved fiber tips. The design is modeled using PhotonDesign software modules (FIMMWAVE and FIMMPROP), which utilizes a fully vectorial finite-difference mode solver and a beam propagation method. The optical properties of single mode pure silica Rad Hard optical fiber (Nufern: S1550-HTA) with core refractive index of 1.45735 and cladding refractive index of 1.44715 at 1550 nm wavelength are used to represent the fiber. The measured complex refractive indices of in-house synthesized Ge-Se and Ge-S compositions in amorphous and crystalline phases (Table 1) from [33] are imported into the simulation models. Table 1 summarizes the T${}_{g}$, T${}_{o}$, and T${}_{c}$ for all synthesized and studied compositions. Among the big ChG family, Ge-Se and Ge-S systems were chosen in the proposed device because (1) these compositions are thermally stable within the range of 400 to 650 °C and (2) the glass-forming regions of these systems are quite extensive, assuring a significant number of compositions to work with. The graphs representing the refractive index values as a function of temperature are accessible in [33].

Among all glassy phases in both systems, three types of compositions have been chosen for detailed characterization chalcogen rich glasses containing 30 at % Ge, stoichiometric compositions (GeSe(S)${}_{2}$) with 33 at % Ge and Ge rich compositions containing 40 at % Ge. These are the most characteristic representatives of these two systems, whose structure, based on the valence requirements of the participating atoms, is characterized with Ge-Chalcogen tetrahedra in which Ge atom is in the center of the tetrahedron surrounded by 4 chalcogen atoms [34]. These tetrahedra can be connected to each other with their corners, forming corner-sharing structural units, or with their edges, forming in this manner edge-sharing units [35]. The other member of this structure is chalcogenide chains in which the chalcogen elements are twofold coordinated. The Se-Se chains in the Ge–Se system are usually situated within the tetrahedral structure and connect the tetrahedral structural units, while in the Ge-S system the S-S units usually form 8-member rings. They are phase separated from the main tetrahedral structure. Chalcogen chain structures are characteristic for the chalcogen rich compositions and they are not supposed to occur in the stoichiometric compositions. However, due to the disordered character of the glass structure, they are available in some cases and are usually considered as “wrong Se(S)–Se(S) bonds”. This respectively leads through lack of chalcogens to formation of “wrong” Ge-Ge bonds [36]. Indeed, the number of the chalcogen–chalcogen and Ge–Ge wrong bonds are the same, which corresponds to the broken chemical order in the glasses. In the Ge rich compositions the Ge-chalcogen tetrahedra forms the structure, similar to that of ethane, which is just called ethane-like. In this structure, two Ge atoms are connected to each other and each has three more chalcogen neighbors for satisfying the Ge atoms valence requirements [37].

### 2.1. Determination of the Optical Constants of In-House Synthesized ChGs

The optical properties of each of the in-house synthesized ChGs were measured using the following method: First, on a silicon substrate, a thin film of the composition under study was thermally deposited. Next, ellipsometry (J. A. Wollam’s M-2000) with in situ heating using an enclosed ellipsometer-compatible hot stage (THMSEL600 by Linkam Scientifc) was performed to obtain the refractive indices (n) and extinction coefficients (k) from the film. An example of the measured refractive index (n) and extinction coefficient (k) for a Ge${}_{40}$Se${}_{60}$ composition as a function of temperature at 1550 nm wavelength is shown in Table 2. It can be seen that the refractive index (n) and extinction coefficient (k) are very different in the crystalline and the amorphous phases.

### 2.2. Fiber Sensor Modeling

**Figure 2:**
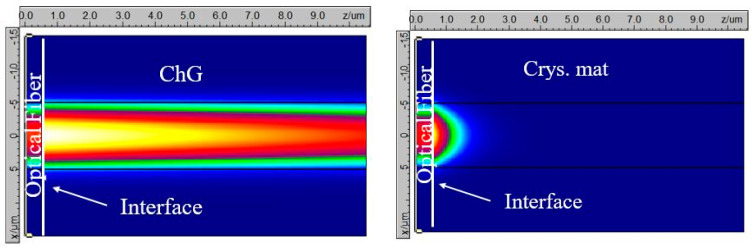
Ge_40_Se_60_-capped fiber device: Power distribution at the fiber:ChG interface for (**a**) amorphous phase and (**b**) crystalline phase.

In the amorphous phase, ChG behaves like a dielectric material with a lower absorption coefficient compared to that in the crystalline phase. From Table 1, it is evident that compared to the amorphous phase, the complex refractive index of ChG in the crystalline phase is vastly different. Thus, the intensity of the reflected light back into the fiber from the ChG-fiber tip interface is also expected to be different. This forms the basis of our sensor’s operating mechanism. For example, the simulated result in Figure 2a shows the transmitted power in Ge${}_{40}$Se${}_{60}$-capped fiber device in the amorphous phase. It can be seen that due to a low absorption coefficient and a modest difference in the refractive index between ChG and fiber mode, most of the light transmits through the ChG. When the temperature is higher than the crystallization temperature of ChG (T > T${}_{c}$), the material crystallizes. In this phase, the Ge${}_{40}$Se${}_{60}$ has higher refractive index, as well as a higher extinction ratio coefficient (behaves like a metal). Thus, a higher fraction of light is reflected back into the fiber, and the transmitted power inside the ChG decays rapidly into the material, as shown in Figure 2b. This change in the reflected power level occurs at very well-defined temperatures (∼T = T${}_{c}$), monitoring of which provides information regarding the node temperature. Similarly, the effect of all the other ChG composition’s refractive index profiles in their amorphous and crystalline phases on the reflected power level can be explained.

**Figure 3:**
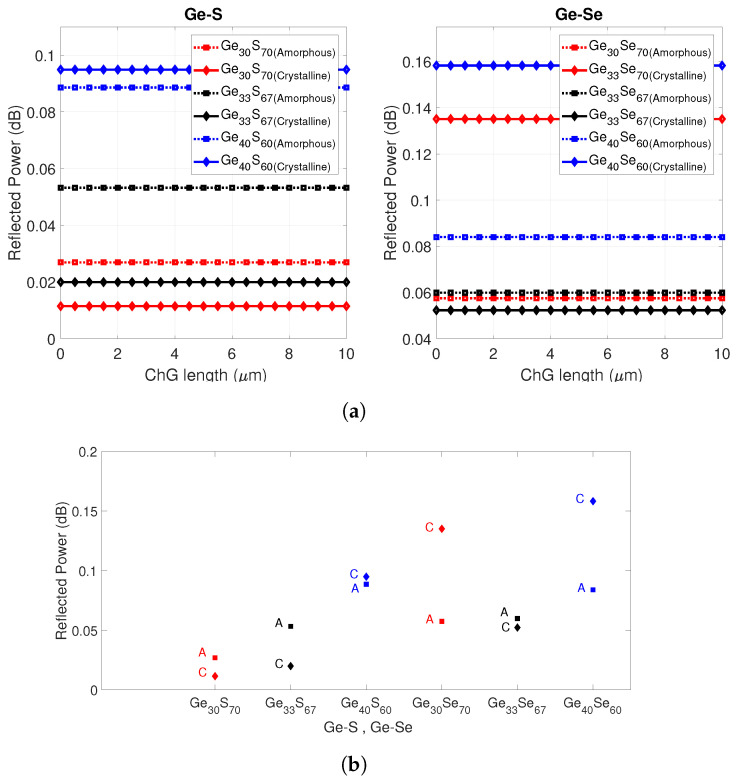
(**a**) Normalized reflected power of the fibers capped with in-house synthesized Ge-S (left) and Ge-Se (right) compositions. The solid curve indicates crystalline phase and the dashed-dotted curve indicates amorphous phase. (**b**) Normalized reflected power of Ge-Se- and Ge-S-capped fiber tips in amorphous and crystalline phases.

Next, the parametric effect of ChG material cap thickness on the reflected powers in the device was studied. Figure 3a shows the normalized reflected power back into the fiber for Ge-S (left)- and Ge-Se (right)-capped fiber devices, respectively, as a function of ChG cap thickness. The reflected power into the fiber shows different power level in the crystalline phase and amorphous phases due to the different extinction coefficient of the material as shown in Figure 3b. For most of the studied compositions, selecting an end cap thickness less than 10 μm provides a relatively high extinction ratio that can be easily detected. From the simulation results, it is observed that all compositions except Ge${}_{33}$S${}_{67}$, Ge${}_{33}$Se${}_{67}$, and Ge${}_{30}$S${}_{70}$ show a considerable difference in extinction coefficient; examples are Ge${}_{40}$S${}_{60}$, Ge${}_{40}$Se${}_{60}$, and Ge${}_{30}$Se${}_{70}$. All compositions (except Ge${}_{33}$S${}_{67}$, Ge${}_{33}$Se${}_{67}$) crystallize by a heterogeneous process by which several different crystalline phases appear and the structure becomes much denser, which leads to an increase in the refractive index [33]. The stoichiometric compositions, Ge${}_{33}$S${}_{67}$ and Ge${}_{33}$Se${}_{67}$ have similar total reflected power in both phases, which indicates that small changes occur in the material structure and band gap during the crystallization process, which is homogeneous in its nature [33].

## 3. Sensor Fabrication and Testing

The proposed sensor is fabricated using a radiation hardened pure fused silica core gold-coated fiber (FiberGuide AFS50/125/155G). The length of the employed fiber is about 50 cm. The fabrication process involves two steps. In the first step, to ensure a cleaved fiber tip, about 3–4 cm of gold from the fiber end was stripped by immersing the tip in Aqua Regia solution for 5–10 min [38]. In the second step, the exposed fiber tip was cleaved using a standard fiber cleaver and the cleaved tip was coated with ChG. The effects of two coating methods to fabricate the sensors were studied: (a) dip coating and (b) thermal evaporation. While dip coating relies on forming a ChG ink, thermal evaporation method is a standard process and provides a baseline for comparison. The fabrication process is as follows.

(a) Dip-coating method: The sensor devices were made by dip coating the fiber in ChG nanoparticle ink [39,40]. The coatings were left to dry for 24 h at room temperature. Then, the coatings were further cured using a hot chuck in a two-step process: (1) the coated fiber was heated at 100 °C for 2 h to slowly dry the solvent, cyclohexanone, without creating cracks in the film, and (2) the fiber tip was placed on a hot plate and heated at 350 °C for 15 min to decompose the surfactants in the ink, Ethyl Cellulose. Once cooled, the fiber tip was dip-coated with spin-on-glass for isolation of the sensor from an oxygen containing ambient. After drying at room temperature for 24 h, the coated fiber was heated at 300 °C for 3 h to cure the spin-on-glass.
(b) Thermal evaporation: The ChGs on the tip of the fibers were also coated using thermal evaporation in a Cressington 308R coating system at 10${}^{-6}$ mbar vacuum with an evaporation rate of 0.35 Å/s. The fiber was not heated during the film preparation. The thickness of the deposited film was estimated using the output from a quartz crystal microbalance. To check the composition of the deposited coating, ChG was also deposited on a single crystalline silicon substrate along with the fiber. Compared to the composition of the source material, the thin film had ±1.5% compositional deviation as measured by an Energy Dispersive Spectroscopy (EDS) study. Once the deposition was completed, the fiber tip was dip-coated with spin-on-glass. After drying at room temperature for 24 h, the coated fibers were heated at 300 °C for 3 h to completely cure the spin-on-glass. This vapor phase deposition process to form ChG layer on fiber tip is a standard process that leads to highly conformal and uniform in thickness coating. The thermally deposited sensors were used to benchmark the performance of the dip-coated devices.

## 4. Experimental Setup

**Figure 4:**
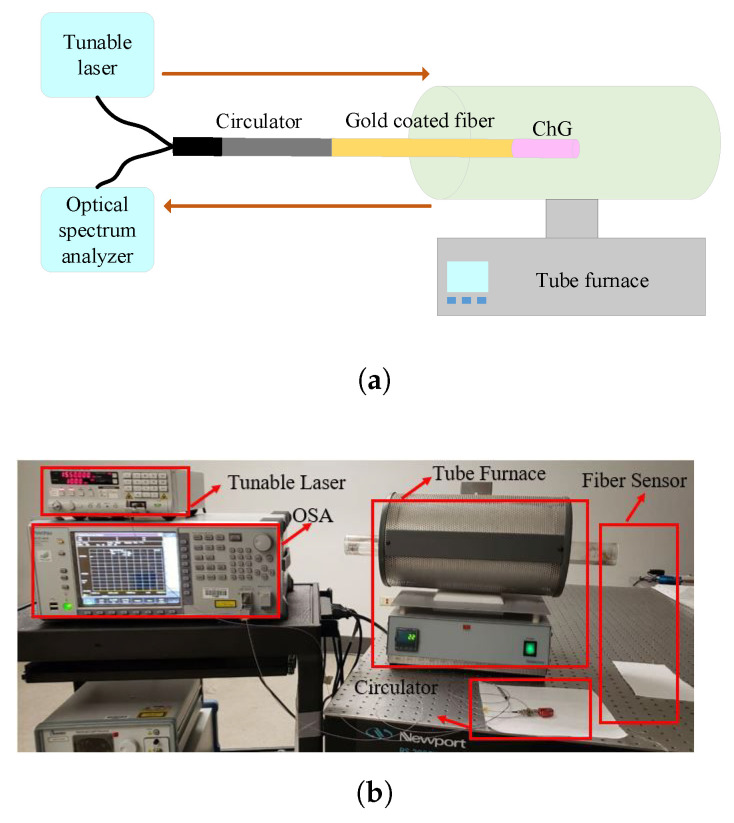
(**a**) Schematic of setup for testing the temperature performance of the fabricated sensors and (**b**) a picture of the actual device characterization setup.

The performance of the fabricated sensor devices was characterized using the experimental setup shown in Figure 4. A 1550 nm wavelength light from a tunable laser source was injected into the fiber sensor through a circulator. The light power reflected from the fiber sensor was analyzed using an optical spectrum analyzer (Anritsu MS9740A). The ChG-capped fiber tip itself was placed inside a high temperature-controlled tube furnace (Eurotherm 2116 controller). The furnace temperature was increased from room temperature (∼25 °C) and 650 °C in 10 °C/min steps. For evaluation of the real- time response of the sensor, the temperature inside the furnace as a function of the time was tracked as well.

## 5. Results and Discussion

**Figure 5:**
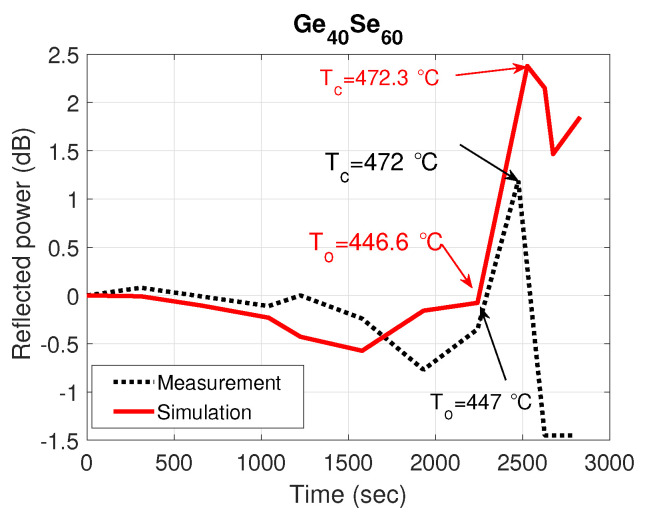
Simulated and measured normalized reflected power as a function of time with Ge${}_{40}$Se${}_{60}$-capped fiber tip.

**Figure 6:**
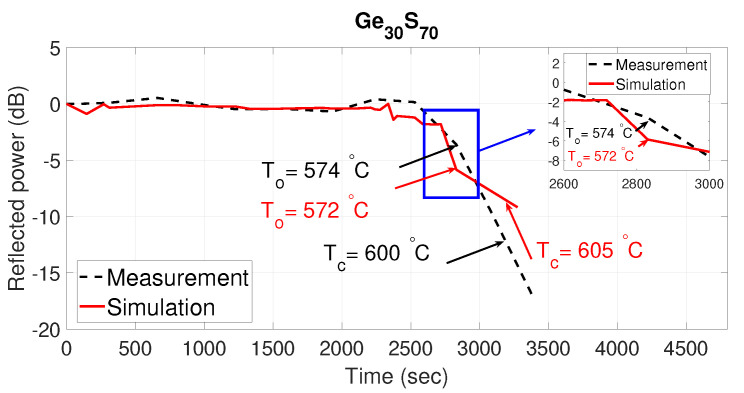
Simulated and measured normalized reflected power as a function of time with Ge${}_{30}$S${}_{70}$-capped fiber tip.

**Figure 7:**
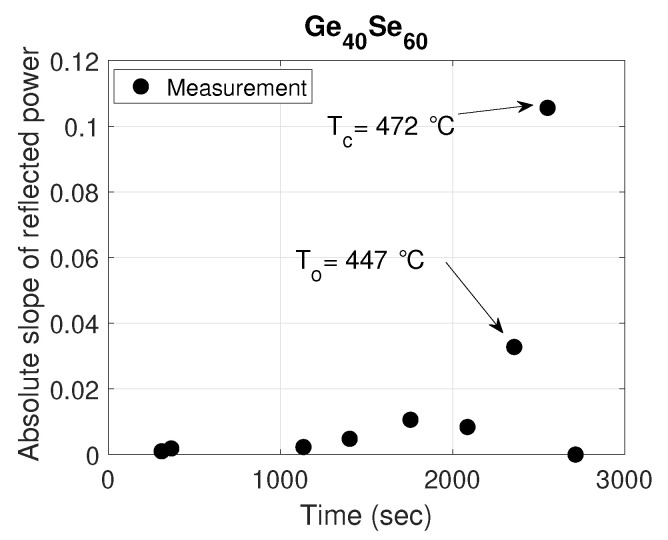
Temperature response of evaporated Ge${}_{40}$Se${}_{60}$-capped fiber-tip-based temperature sensor.

The normalized measured reflected power (dotted black curve) as a function of time for two compositions: Ge${}_{40}$Se${}_{60}$ and Ge${}_{30}$S${}_{70}$ are shown in Figure 5 and Figure 6, respectively. The normalized simulated reflected power with extracted refractive index profile from studying the particular ChGs as a function of time is also plotted (solid red curve). It can be seen from the figures that the measured and the simulated results match very well. As the proposed sensor works on the principle of the phase change of ChG material, which is highly temperature dependent, abrupt changes in the reflected power are observed, as expected. These sudden changes and associated temperatures can be efficiently extracted from the sensor data by plotting the slope as a function of time. For example, from the data regarding the Ge${}_{40}$Se${}_{60}$ composition shown in Figure 5, a plot of the slope of measured reflected power as a function of time is shown in Figure 7. This figure manifests a big growth of the absolute slop of the reflected power between the T${}_{o}$ and T${}_{c}$ due to the large structural reorganization occurring in a solid state from the moment of the occurrence of the first crystallizes at the onset of crystallization T${}_{o}$ up to the full crystallization of the material at T${}_{c}$. Two peaks stand out at 2462 s and 2627 s, corresponding to the onset and peak crystallization temperatures of 447 °C and 472 °C obtained from monitoring the temperature inside the furnace, which is close to the predicted temperatures of 446.6 °C and 472.3 °C.

The data related to the onset and peak crystallization temperatures calculated using the slope method for all studied compositions are shown in Table 3. According to the measured refractive index data presented in Table 1, two crystallization temperatures for Ge${}_{40}$S${}_{60}$ are expected. The experimental data, however, only provide one of the crystallization temperatures (485 °C) as shown in Table 3. This is because at the lower crystallization temperature, the structure of the material is not strongly organized and thus no strong optical changes are observable. Table 3 also provides the expected temperatures error in the measured and the expected T${}_{c}$. It can be seen that except from the evaporated Ge${}_{33}$Se${}_{67}$ and dip-coated Ge${}_{30}$Se${}_{70}$ samples, all other samples are in good agreement within ±0.4–2 °C. The absolute slop of the reflected power decays because of cracks formation during the crystallization process and increase of the interface roughness. These effects can be overcome by encapsulation of the devices and/or melting of the films and their solidifying in amorphous condition during the reversing process for reusage of the devices.

Based on our preliminary detailed studies of these materials and the collected Raman spectroscopy data, we suggest that this is a result of the fact that the stoichiometric composition has very homogeneous structure in the amorphous condition with low number of wrong bonds and this structure is maintained upon crystallization as well [33]. Similarly, the glasses with composition Ge${}_{30}$Se${}_{70}$, which have very close structure to the one of the stoichiometric composition Ge${}_{33}$Se${}_{67}$, perform with a small difference of the optical properties after crystallization due to the lack of strong reorganization of their structure. This is not the case for the Ge${}_{30}$S${}_{70}$ compositions, in which the 8-member S rings open up at higher temperatures to become a part of the tetrahedral backbone of the crystalline material, thus leading to observable change in the refractive index of this material. The crystallization kinetics and the formation of different structural units in these glasses are discussed in detail in [33,41,42].

In addition to the change in density, it has been argued [36] that the optical and electrical property contrast due to phase change originates mostly from transformation in the structural medium-range order after crystallization. The crystalline GeSe(S)${}_{2}$ that appear after the phase change have a predominantly corner-shared structure and are pseudo-two-dimensional when the low temperature forms of these materials crystallize [43,44]. In the high-temperature dichalcogenides, the corner-sharing units are connected with edge-sharing building blocks [45]. Although, the kinetics and materials are studied extensively, the change in optical properties due to crystallization is not well understood in these compositions. For example, except for x = 40, Ge-S compositions show a decrease in refractive index after crystallization. We suggest that this is due to one more detail related to the Ge${}_{40}$Se(S)${}_{60}$ studied compositions—in accordance with the XRD studies besides GeSe(S)${}_{2}$, the crystalline form occurring after the phase change contains also GeSe [33] which has orthorhombic structure [46]. In this structure, both atoms are threefold coordinated due to occurrence of dative bonds. It is for this reason that the Ge-rich compositions react with bigger changes in optical properties after crystallization. In Ge${}_{33}$Se${}_{67}$, the change in optical properties due to crystallization is not distinct due to lack of medium range structural changes. For Ge${}_{30}$Se${}_{70}$, some previous studies [47] reported a reduction in refractive index which is also the case for the examined thin films until their temperature reaches the crystallization temperature. These data are from ellipsometric studies of thin films on flat substrates, but the fiber devices showed an increase in reflected power after crystallization, which is direct evidence of the fact that the refractive index increases after crystallization of Ge${}_{30}$Se${}_{70}$. We assume that in the case of fiber there are additional interference effects occurring which influence the results measured from the fiber devices.

## 6. Temperature Profile Estimation Using Array Sensor

**Figure 8:**
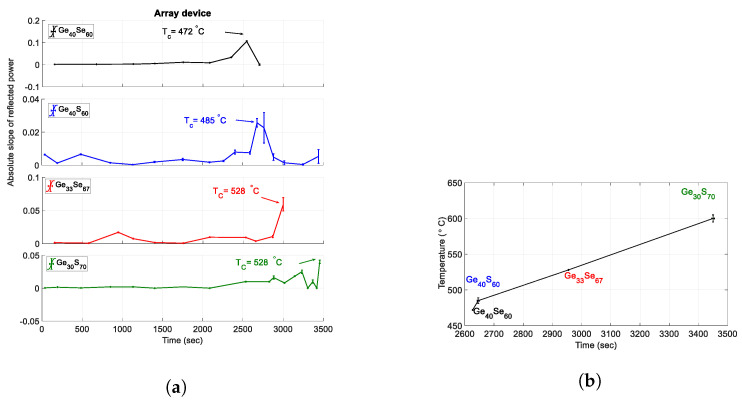
(**a**) Temperature response of the sensor array and (**b**) monitored temperature trend as a function of time using array structure.

Arranging the single ChG tip-coated fibers in an array structure and monitoring the reflected power from each fiber will help with the real-time detection of several temperatures inside extreme environments, thus allowing mapping out the temperature profile. As mentioned before, each of the synthesized ChG compositions has a specific crystallization temperature which allows for accurate monitoring and recording of real-time temperature profile within a desired environment. As an example, the slope of the reflected power data from the array structure comprising of four optical fibers capped with four different ChGs (Ge${}_{40}$Se${}_{60}$, Ge${}_{30}$S${}_{70}$, Ge${}_{40}$S${}_{60}$, and Ge${}_{33}$Se${}_{67}$) within a temperature range of 472 °C to 600 °C is shown in Figure 8a. At lower temperature below T${}_{o}$ there are structural fluctuations in the solid state, due to the initial stages of crystalline organization of the material. For a better reading, the measurements presented at temperatures above T${}_{o}$ are demonstrated at higher resolution. The proposed array sensor is arranged from ChG dip coated devices which show lower error in temperature response. Correlating the peak slope and times provides a temperature evolution chart as shown in Figure 8b, while four distinct temperatures, 472 °C, 485 °C, 528 °C, and 600 °C are recorded with this array structure. Increasing the array size to accommodate several other compositions of ChGs will enable real-time and precise monitoring of temperature with higher temperature resolution. As the fiber diameter is 125 μm, an array of 15–20 sensors, leading to a temperature resolution less than 10 °C within 440–600 ${}^{{}^{\circ}}$C will result in the sensor node area size smaller than 2.125 mm${}^{2}$, which makes this array easily deployable within structures.

## 7. Conclusions

A novel fiber-optic sensor was modeled and demonstrated for monitoring the temperature within extreme environments. The working principle of the sensor depends on the phase transition of the ChG deposited on optical fiber tip from amorphous to crystalline phase at well-defined temperatures, thereby causing rapid changes in the complex refractive index of the material. These changes in the optical properties lead to sudden changes in the reflected intensity. Using six different compositions of in-house synthesized ChGs, a sensor array performance is demonstrated that can effectively track temperature from 440 to 600 °C. These optical fiber sensors promise a very reliable, small size, lightweight sensor network for use within high-temperature and high-radiation environments. Studies of this material reveal that the occurrence of big compositional and structural changes in Ge-rich compositions after crystallization lead to better expressed devices effects, which is important when considering material choices for building devices. These compositions offer phase change temperatures close to those of chalcogen rich materials. The suggested devices are reusable after application of electrical field at room temperature over the ChG covered fiber tip, which creates Joule heating that melts the ChG. The melt solidifies in amorphous condition due to a fast cooling from the fiber core which has much bigger volume than the ChG film.

## Figures and Tables

**Table 1:** Summary of the measured complex refractive indices of synthesized glasses in amorphous and crystalline phases at 1550 nm wavelength.

Composition	Refractive Index		Temperature (°C)		
	Amorphous	Crystalline	T${}_{g}$	T${}_{o}$	T${}_{c}$
Ge${}_{30}$S${}_{70}$	2.17406 + i0	1.77269 + i0.11865	402	572	605
Ge${}_{40}$S${}_{60}$	2.67680 + i0	2.72309 + i0.17664	355	408	413
				480	489
Ge${}_{33}$S${}_{67}$	2.31779 + i8.28 $\times 10^{-6}$	1.92455 + i0.02458	435	644	694
Ge${}_{30}$Se${}_{70}$	2.37646 + i4.06 $\times 10^{-5}$	3.12455 + i0.25837	334.8	440.9	470.4
Ge${}_{40}$Se${}_{60}$	2.63104 + i0.00575	3.10991 + i0.211	343.7	446.6	472.3
Ge${}_{33}$Se${}_{67}$	2.38753 + i0.00402	2.30756 + i0.02011	396.3	485.4	527.7

**Table 2:** Measured complex refractive index of Ge${}_{40}$Se${}_{60}$ at different temperatures at 1550 nm wavelength.

Temperature (°C)	Refractive Index	Temperature (°C)	Refractive Index
25	2.717 + i0.00547	400	2.70088 + i0.01636
100	2.171516 + i0.00575	450	3.35909 + i0.25735
150	2.695050 + i0.00547	472	3.29764 + i0.09341
200	2.66978 + i0.00513	479	3.1099 + i0.2211
250	2.63104 + i0.00575	484	3.14107 + i0.22572
300	2.59792 + i0.00563	500	3.2688 + i0.21606
350	2.70057 + i0.00938		

**Table 3:** Temperature response of Ge-S and Ge-Se tip-coated optical fiber-based temperature sensor.

Composition	Fabrication	T${}_{o(\mathrm{Expected})}$	T${}_{o(\mathrm{measured})}$	Time	T${}_{c(\mathrm{expected})}$	T${}_{c(\mathrm{measured})}$	Time	Error
		(°C)	(°C)	T${}_{o(\sec)}$	(°C)	(°C)	T${}_{c(\sec)}$	T${}_{c}$ (°C)
Ge${}_{40}$Se${}_{60}$	Dip-coated	446.6	460	2589	472.3	472	2627	0.3
	Evaporated	446.6	447	2462	472.3	472	2627	0.3
Ge${}_{33}$Se${}_{67}$	Dip-coated	485.4	485	2646	527.7	528	2950	0.3
	Evaporated	485.4	450	2527	527.7	485	2646	42.7
Ge${}_{30}$Se${}_{70}$	Dip-coated	440.9	400	2241	470.4	450	2527	20.5
	Evaporated	440.9	447	2462	470.4	460	2589	10.4
Ge${}_{40}$S${}_{60}$	Dip-coated	480	450	2527	489	485	2646	4
Ge${}_{30}$S${}_{70}$	Dip-coated	572	574	2830	605	600	3399	5

## Data Availability

Data sharing not applicable.

## Abbreviations

The following abbreviations are used in this manuscript:

ChG	Chalcogenide Glass
RTD	Resistance Temperature Detectors
OFS	Optical Fiber Sensors
T${}_{g}$	Glass Transition Temperature
T${}_{o}$	Crystallization Onset Temperature
T${}_{c}$	Glass Peak Crystallization Temperature
DSC	Differential Scanning Calorimetry
SMF	Single-Mode Fiber
